# Relationship and New Prospectives in Joint Hypermobility in Children with Autism Spectrum Disorder: Preliminary Data

**DOI:** 10.3390/jpm13121723

**Published:** 2023-12-18

**Authors:** Domenico Marco Romeo, Marianna Moro, Mariangela Pezone, Ilaria Venezia, Federica Mirra, Margherita De Biase, Agnese Polo, Ida Turrini, Maria Rosaria Lala, Chiara Velli, Francesca Sini, Domenico Dragone, Eugenio Mercuri, Claudia Brogna

**Affiliations:** 1 Pediatric Neurology, Università Cattolica del Sacro Cuore, 00168 Rome, Italy; domenicomarco.romeo@policlinicogemelli.it (D.M.R.); mariannamoro@libero.it (M.M.); m.pezone01@gmail.com (M.P.); venezia.ilaria@gmail.com (I.V.); fedemirra20@gmail.com (F.M.); margherita.debiase@gmail.com (M.D.B.); agnesepolo1993@gmail.com (A.P.); eugeniomaria.mercuri@policlinicogemelli.it (E.M.); 2 Pediatric Neurology Unit, Fondazione Policlinico Universitario “A. Gemelli”, IRCCS, 00168 Rome, Italy; ida.turrini@guest.policlinicogemelli.it (I.T.); chiara.velli@tiscali.it (C.V.); francsini@hotmail.it (F.S.); 3 Neuropsychiatric Unit ASL Avellino, 83100 Avellino, Italy

**Keywords:** join hypermobility, autism spectrum disorder, cognitive assessment

## Abstract

Autism spectrum disorder (ASD) and joint hypermobility (JH) are considered two different etiological and clinical entities that most often appear in childhood. Despite growing increased research showing a co-occurrence for both conditions, a link between them is rarely established in clinical settings, and the relationship between ASD and JH has not so far been completely investigated in all age groups of ASD children. This preliminary study examined a cohort of 67 non-syndromic ASD children aged 2–18 years (sex ratio M:F = 12:1) showing different degrees of cognitive impairment and autism severity, using the Beighton scale and its revised version. A total of 63% of ASD patients aged 2–4 years and 73% of ASD patients aged ≥5 years presented significant scores of hypermobility. No significant correlation was found comparing total laxity score and cognitive assessments and severity of autistic symptomatology (*p* > 0.05). The results suggest that JH could be considered as a clinical characteristic of ASD patients and it needs to be assessed in order to schedule a better rehabilitation program.

## 1. Introduction

In the last few years, more attention has been focused on joint hypermobility (JH), both in adult and pediatric populations. Joint hypermobility or joint laxity is defined as a more-than-normal range of movement (ROM) in a single joint or a group of joints [1]. From a clinical point of view, the impact of JH might differ significantly on the basis of age at assessment, body distribution, and aetiology. When JH is observed in one or a few types of joints (usually less than five), it can be defined as localized joint hypermobility (LJH). LJH may be inherited, but it may also be an acquired trait related to trauma, joint diseases, or other conditions [2]. JH in multiple sites (usually five or more) may be defined as generalized joint hypermobility (GJH) [2]. It is also seen more often in Asian and African ethnicity compared to Caucasians [3]. JH can exist as an isolated diagnostic finding, but it could be a feature of a larger syndromic diagnosis and it is also a unifying feature in the heritable disorders of connective tissue (HDCTs) including the Ehlers–Danlos syndrome, Marfan syndrome, and osteogenesis imperfecta in which hypermobility is generally associated with other clinical features [4].

In the majority of cases, however, joint hypermobility is observed in the clinic as an isolated phenomenon, defined as asymptomatic hypermobility; in some cases, it is also associated with musculoskeletal symptoms such as arthralgia, pain, and extraarticular manifestations (skin hyperextensibility, tendency for osteopenia, gross motor developmental delay), in the absence of other heritable disorders of connective or muscle tissue or other causes of the symptoms [1,2,5].

General population studies in children have shown a prevalence of 10–15% in boys and 20–40% in girls aged 11–17 years, comparable to those in adults, and between 5–30% at lower ages [3,4]. It has been found that JH diminishes with age from childhood onward and it is about three times more common in females than males [3,4,5,6].

Children with joint hypermobility can show a high level (30–50%) of coordination difficulties and other neurodevelopmental disorders. A few papers have suggested a link between JH and selected neurodevelopmental disorders; others have explored the neurodevelopmental profile of children with hereditary connective tissue disorders (HCTDs) and developmental coordination disorder including motor, cognitive, executive-attentive, and emotional-behaviour features [7,8]. Moreover, speech and language disorders, poor writing skills, and attention deficit-hyperactivity disorder might be frequent in subjects with GJH with an impact on academic performances [7]. This suggests the existence of a link between an abnormal connective tissue and an abnormal neurodevelopmental outcome in the absence of a central pathway that impact on cognitive development [7], possibly due to a defective proprioception and/or impaired vestibular system.

In the last few years, different research articles have proposed a possible link between hypermobility related disorders (HRDs) and autism spectrum disorder (ASD), suggesting that these co-occur more often than expected by chance [7,8].

ASD is a severe neurodevelopmental disorder defined by DSM-V A and B criteria as the presence of persistent deficits in social communication and social interaction across multiple contexts and the presence of restricted, repetitive patterns of behavior, interests, or activities [9]. About 85% of individuals with ASD have the so called “idiopathic” ASD, determined by the interaction between epigenetic and environmental factors (many of which are still unknown) influencing the optimal neurodevelopment of the child, while about 15% are diagnosed with “secondary” ASD, determined by underlying genetic conditions [9].

The prevalence of this disorder is high, recently up to 1/54 children, representing a significant public health problem. The disturbances caused by ASD are present from early infancy and cause clinically significant deterioration in crucial areas of a child’s functioning [9]. Many genetic and environmental contributors to the pathogenesis of ASD have been identified, also resulting in great interindividual variability at the clinical level [10].

Few studies have already shown the association between ASD and joint hypermobility in adults [11,12]. Children with ASD can show motor impairments, like difficulties in gait, balance, coordination, fine and gross motor skills, and sensory–motor integration. It has been hypothesized that these problems could be due to brain damage, deficiency in motor control, and motor learning, and indirectly to muscle weakness, hypotonicity, hypermobility, and ligament laxity. Hypermobility is also related to pain and proprioceptive deficits, that often ASD children are not able to express, due to their communication difficulties [13]. In the developing child with joint hypermobility, a generalized lack of proprioception that may affect the process of organization of spatial and temporal concepts has also been proposed. These circuits are most often reported to be involved in ASD in both neuroimaging and electrophysiological studies [14].

Assessing JH is not always easy due to different influencing factors, such as age, gender, and ethnicity [7]. In the past years, several tools were elaborated on for the clinical evaluation of the JH. The Beighton nine-point scoring system, based on the analysis of the ROM of all major joints, is widely used as a clinical screening test for analyzing joint hypermobility in children [1,14]. It was initially validated and used in children from 5 years of age, and more recently adapted for children younger than the age of 5 years (12–60 months) [1,15]. The hypothesis of the present study is that non-syndromic children with ASD could have a higher incidence of JH compared to typically developed children with a possible correlation between the cognitive profile and the severity of ASD symptoms.

Therefore, the primary aim of this preliminary study is to (1) evaluate the presence of JH in a group of non-syndromic ASD children by using the Beighton score and its revised version; (2) the secondary aim is to identify the possible relation between the level of JH and the severity of ASD also including the cognitive profile.

## 2. Material and Methods

This study is an observational retrospective monocentric study, conducted in the Pediatric Neurology Unit of the Fondazione Policlinico Agostino Gemelli Hospital, IRCCS, in Rome, Italy, from January 2017 to July 2022.

Patients meeting the following inclusion criteria were enrolled in the study:

- Age between 2 and 18 years old;
- A clinical diagnosis of ASD according to the DSM-V criteria [9];
- The presence of at least one cognitive evaluations, according to their chronological age.

The study was approved by the ethics committee of our Institution (ID: 3418, prot. N. 0037324/20). Informed consent was obtained from parents of the children included in the present study. The investigations were carried out following the rules of the revised Declaration of Helsinki.

All the children performed a brain magnetic resonance imaging (MRI) and a genetic assessment (array comparative genomic hybridization (a-CGH)) at the time of the diagnosis. Children with a diagnosis of genetic syndromes or metabolic diseases and brain malformations were excluded from the study.

### 2.1. ADOS-2 Assessment

All patients underwent ADOS-2 assessment in order to identify the severity of the disease [16].

The ADOS-2 is a semi-structured, standardized observational tool created to evaluate and identify ASD in individuals of all ages. The ADOS was created in 1989 and was revised into the ADOS-2 to increase the assessment’s precision and adaptability. The ADOS-2 updated the classification algorithms, changed the administration procedures, included a module for toddlers between the ages of 12 and 30 months, and established new standards for comparison scores that permit the analysis of the severity of ASD symptoms across different modules. The ADOS-2 consists of five modules, each of which contains activities based on children’s expressive language proficiency and developmental age. The widespread use of this test may be due to its capacity to collect data from a series of structured activities, to detect autistic behaviors during interactive activities, and to take into consideration a variety of developmental stages and ages. The ADOS-2, indeed, incorporates semi-structured and unstructured tasks designed to elicit play, repetitive and restrictive behaviors, and non-verbal and social communication from the patient. Currently, the ADOS-2 has been translated into more than 20 languages and its clinical validity has been demonstrated in numerous international samples [17]. To be classified as having an ASD using the ADOS-2, a person’s score must meet or exceed the cut-off for both the social affect and repetitive and restrictive behavior domains. Based on the total score obtained, patients fall into three categories: autism, autism spectrum, or non-autism. The comparison score identifies the severity of symptoms, with a value from 0 to 10, increasing according to gravity.

### 2.2. Cognitive Assessments

All patients performed non-verbal and verbal skills including the following:

- Griffith Mental Development Scale (GMDS): is a widely used test to measure young children’s development from birth to the age of eight. The GMDS consists of six separate subscales: Locomotor (A), Personal-Social (B), Language (C), Eye–Hand Coordination (D), Performance (E), and, for children aged 2–8 years, Practical Reasoning (F) [18].
- The Wechsler Preschool and Primary Scale of Intelligence-Third Edition (WPPSI-III) is divided into two age groups, the younger group covering the ages of 2.6–3.11 and the older group covering the ages from 4.0 to 7.3. It provides an estimate of the cognitive profile measured as total IQ ad, for both age bands, and there are scores for the Performance IQ, Verbal IQ (VIQ), and General Language Composite (GLC). Only for the older age band is there also a subscale for Processing Speed (PSQ) [19].
- The Wechsler Intelligence Scale for Children-Fourth Edition (WISC-IV) evaluates intellectual ability of children from 6 to 16 years and 11 months. It provides an overall measure of general cognitive ability, and also of intellectual functioning in Verbal Comprehension (VC), Perceptual Reasoning (PR), Working Memory (WM), and Processing Speed (PS) [20].
- Leiter International Performance Scale-Revised (Leiter-R) is a personally administered IQ test for people between the ages of 2 and 20 years and 11 months. Since neither the examiner nor the child must speak, it is purely non-verbal [21].

### 2.3. Joint Hypermobility Assessment

The ligament laxity of every patient enrolled in this study was evaluated according to the age of the patients measured with the Beighton Score [1] for children from 5 to 18 years of age, and with the Revised Beighton score [15] for children with age between 12 months and 4 years and 11 months. Therefore 2 different groups aged related were considered.

The Beighton score is a scale used to estimate the presence of joint hypermobility that has also been evaluated in children older than 5 years old. It consists of the following five items: (1) bilateral passive dorsiflexion of the fifth metacarpophalangeal joint score (positive if ≥90°); (2) bilateral passive hyperextension of the elbow (positive if ≥10°); (3) bilateral passive hyperextension of the knee (positive if ≥10°); (4) bilateral passive apposition of the thumb to the flexor side of the forearm, while shoulder is 90° flexed, elbow extended, and hand pronated (positive for joint hypermobility if the whole thumb touches the flexor side of the forearm); and (5) forward flexion of the trunk, with the knees straight (positive if the hand palms rest easily on the floor). A total score ≥4 is considered indicative for ligament laxity [1].

The Revised Beighton score is a revision version of the original Beighton score adapted for children aged younger than 5 years old (range 1 to 4 years). In this scale, the last item of the original score is replaced with the evaluation of passive dorsiflexion of the ankle joint bilaterally (positive when the angle is >30°). A total score >4 is considered indicative for joint hypermobility [15].

The Beighton score was performed in all the children with ASD for the present study and in a population of typically developing Italian preschool and school age children matched for age and sex with no history of neurological or genetic conditions considered as the control group; these children were recruited from nurseries and during routine health clinical assessments at the Pediatric Unit of our Institution.

## 3. Data Analysis

The characteristics of participants are described as mean ± SD. Differences between the two groups of children with ASD according to the age of assessment (< or ≥5 years) and gender for Beighton scores were performed with the Fisher exact test. The comparison between ASD children and the control group for the Beighton Score according to the age of assessment was performed using the Fisher exact test. The correlation between the Beighton score and the ADOS II scores, cognitive score, and subscale score was explored using the Pearson correlation test. The *p* value was set up as <0.05.

Statistical analysis was performed using the R statistical software (version 4.3.0, R Foundation for Statistical Computing, Vienna, Austria).

## 4. Results

A total of 67 children, 62 male (92%) and 5 female (8%) (range: 2–18 years, sex ratio M:F = 12:1), with a mean age 6 years ± 3.87 SD, fulfilled the inclusion criteria. Brain MRI was normal in 80% of the children and reported non-specific features (enlarged anterior ventricular horns and/or lateral ventricle areas, reduced total and/or posterior callosal sub-region areas) in the remaining 20%. Array CGH reported normal results in all the children. More details including age and total IQ and ADOS II score are reported in Table 1. More details including Beighton score are reported in Table 2.

The other 67 typically developing children, 62 male (92%) and 5 female (8%) (range: 2–18 years, sex ratio M:F = 12:1), with a mean age 6 years ± 3.84 SD, were also assessed using the Beighton score and considered as the control group. Details of the results of this group are reported in Table 3.

A total of 19/30 (63%) ASD patients aged 2–4 years presented a Beighton total score >4, and 27/37 (73%) of ASD patients aged ≥5 years presented a total laxity score ≥4.

Statistically significant differences (*p* < 0.05) were observed between the means of the total score of the clinical sample and the normative sample of both age groups (<5 years and >5 years) for the Beighton global score and the single item.

For preschool age children, in the individual items, a score of 1, indicating joint laxity, was found more frequently in three items: “apposition of the thumb to the forearm”, “passive dorsiflexion of the fifth finger”, and “passive hyperextension of the elbow” (63%, 75%, and 65%, respectively). In the other two items, “passive hyperextension of the knee” and “passive dorsiflexion of the ankle”, a score of 1 was found in 30% and 43% of the population, respectively. For children ≥5 years, a score of 1, indicating joint laxity, was found more frequently in three items: “apposition of the thumb to the forearm”, “passive dorsiflexion of the fifth finger”, and “passive hyperextension of the elbow” (48%, 61%, and 68%, respectively). In the other two items, “passive hyperextension of the knee” and “forward flexion of the trunk”, a score of 1 was found in 32% and 38% of the population, respectively.

Scores different from 0 were present among some children in all items at both the ages of assessment, and were bilateral in more than 98% and unilateral in the other 2%.

The Beighton scores were equally distributed according to the age of assessment and gender (*p* > 0.05). No significant correlation was found comparing total laxity score and total IQ or ADOS 2 total score, AS, and CRR scores.

## 5. Discussion

Research in children with ASD has focused mainly on cognitive features, social skills, and emotional aspects, while motor aspects have received little attention. However, in recent years, studies on the motor or sensory–motor behaviors of children with ASD have been explored, evidencing that clinical features of children with ASD as hypotonia, clumsiness, and toe walking are common findings, especially at an early age [22]. Many children with ASD reported static and dynamic postural balance difficulties and diminished postural control affecting locomotion and jumping. More recently, the presence of JH has also been identified in these patients.

Genetic data have suggested similarities at the molecular, cellular, and tissue levels, supported by numerous genetic syndromes characterized by autism and hypermobility with HDCTs in children and adults [11,12,13,14,23]. HDCTs and autism comorbidity and familial co-occurrence give credence to this relationship, suggesting potential connections via the maternal immune system. This relationship also suggests that connective tissue impairment may influence brain development, either through direct and/or indirect causes [7]. Brain abnormalities include a heterotopic formation in the central nervous system [8].

On the other hand, only a few studies have explored the association between non-syndromic JH and ASD children. In general, these children were found to have significantly more supple joints (especially at the metacarpophalangeal and wrist joints) than their typically developing peers [14,23]. However, these studies did not use structured or age-appropriate measures to assess JH.

The present study demonstrated a high incidence of JH measured in a population of preschool age and school age children with ASD. To assess JH, the Beighton score has been used, and is considered as one of the most-used quantitative measures for analyzing joint hypermobility in children from 12 months onwards. Although there is no consensus in the literature on the standardized cut-off points that exist in children, those cut-off points most used in the literature for both age of assessments have been considered [1,15]. Therefore, for the first time, JH has been assessed in a population of young ASD children at least 2 years of age using a structured quantitative measure.

This study found a higher rate of JH in both ASD preschool age (63%) and school age children (73%) than those reported in the literature for typically developing children using the Beighton score at the same age (<10%) [1,15]. These data were also confirmed by comparing the Beighton scores of the ASD population with those obtained in a control group population; a significant higher score in children with ASD in all the items and in the global Beighton score in both preschool and school age groups was observed. The possible etiological mechanism underlying the comorbidity between ASD and JH is not well defined. The presence of a disorder of the connective tissue related to central nervous system abnormalities has been proposed [24,25]. More in depth, subjects with JH reported a significantly greater bilateral amygdala volume than those with no JH and also displayed a decreased volume within other regions implicated in emotional arousal and attention (anterior cingulate, parietal lobe). Moreover, the degree of hypermobility correlated negatively with superior temporal volume, a region implicated in processing social and emotional signals [24]. Differences in amygdala and superior temporal cortex anatomy has been also observed in autism [25]. These data confirm an overlap between both conditions. It has also been suggested that the abnormal signal integration at the level of the circuit involving the amygdala, insula, and anterior cingulate cortex might be responsible for the proprioceptive and emotional imbalances present in ASD [26]. The children included in the present study did not report specific abnormalities in these areas on brain MRI; however, a systematic quantitative/morphological collection of brain MRI was not performed as it was not the aim of the present study; therefore, possible volumetric abnormalities of specific areas in this study sample cannot be excluded.

A further indirect link between ASD and JH could be due to a common alteration in the central nervous system or proprioceptive control; in children with GJH, the presence of hypermobility, dysautonomia, chronic pain, and proprioceptive impairment may influence motor, cognitive, and behavioral skills, and may ultimately affect neurodevelopment [26]. The generalized lack of proprioception, typical of children with JH, may affect the process of organization of spatial and temporal concepts with motor postural and balance control problems [27,28,29,30]; this issue could prevent the optimal acquisition of non-verbal communication skills which may lead to impairment in social interactions typical of ASD [7]. A defective proprioception and/or impaired vestibular system has been recently proposed as a possible link between JH and other neurodevelopmental disorders like developmental coordination disorders, attention deficit-hyperactivity disorder, and learning disabilities; these data seem to confirm that congenital and widespread “laxity” of the connective tissue can directly affect proprioception and vestibular function [8].

Although specific mechanisms responsible for the association between JH and ASD have not been completely elucidated, it is possible to argue that the two conditions may either influence each other (i.e., JH causes ASD or ASD causes JH) or share a common ethiopathogenic process [31].

The analysis of individual items showed that “apposition of the thumb to the forearm”, “passive dorsiflexion of the fifth finger”, and “passive hyperextension of the elbow” had a greater prevalence of joint laxity; these differences are probably due to the larger muscle groups supporting the trunk and the legs, as previously reported in both typically developed infants and the preterm population with different incidence [15,28,29,30]. These data are also in line with a recent study on ASD children, showing a laxity component on the proximal and distal muscles of the upper limbs (wrists and shoulders) and a reduced trunk extensibility due to a physiological hypertonia of the flexor and extensor muscles of the trunk [32]; this clinical pattern may suggest motor anomalies in the cortico-spinal tract or abnormalities in the median descending pathways from the brain stem that regulate the adjustments of the proximal muscles and posture. This tonic component for the muscles of the trunk and the laxity component in the upper limb in children with ASD may represent a phenotype characteristic of these children.

In typically developed children, joint laxity is usually considered to be inversely related to age, especially in the male gender, as younger children show greater joint mobility than older ones [28,29,30], with a greater incidence in females than in males [33]; this is probably due to the high body water content in younger children and in female children and an increase in muscle fibers in older ages, especially in males [29]. In this study population of ASD children, no significant age difference was found, suggesting that JH persisted along the ages; furthermore, a similar incidence of JH in this cohort, regardless of the age of assessment, could be explained by the presence of low muscle tone that is detected more frequently in children with ASD, especially in boys [34]. JH could influence motor development of ASD children due to a reduced proprioception from the joints and reduced strength of the muscles that lead to a poor control of joint movement and instability [35]; in addition, no significant gender difference was noted; however, in this ASD study sample, the incidence of the male gender was very high, typical in children with ASD; therefore, a real comparison between the two genders is not possible.

No correlation between JH and cognition and the severity of ASD was found in either age group; this result confirmed that the presence of JH is a characteristic of ASD not related with the severity of symptoms or cognitive impairment. The degree of hypermobility (according to the Beighton scale) also showed no relation to the degree of associated symptoms like ADHD and ASD [36].

The results of the present study suggest that JH could be considered as a clinical characteristic of ASD children and it needed to be assessed in order to schedule a better rehabilitation program. Since JH is easy to recognize and measure, using the Beighton score from 12 months of age, it is possible to consider it as a reliable early “red flag” for hypotonia [34], in order to suggest an ASD evaluation that could be due to an earlier diagnosis, intervention, and possibly an improved outcome.

JH and hypotonia can impair the ability of the children to explore the environment, and the children could ignore critical visual cues resulting in impaired learning and cognitive development [37]. ASD children with JH could therefore require a different and multilevel rehabilitation approach (neurodevelopmental and cognitive approach, perceptual-motor therapy, motor functional skill approach, appropriate orthotics) [38]; in addition, a prolonged neurodevelopmental follow-up compared to ASD children with no JH is needed. A targeted physical therapy program could improve muscular strength and fitness, including exercises of proprioception, correcting the motion control of joints, balancing manual therapy, and the use of tape, hydrotherapy, and relaxation training [38]. The strength of this study is related to the availability of a complete neuropsychological assessment and the Beighton scores performed in all non-syndromic ASD children included in the study and the long age range involved.

### Limitations of the Study

In considering the validity of the conclusions, the potential effects of some methodological limitations should be considered that may have affected the analysis of the results. Although all the children performed an a-CGH, and dysmorphic features, microcephaly, and/or structural brain malformations were not included in the study sample, it was not possible to exclude the presence of other mutations, including small mutations detected by e exome sequencing analysis included in the ASD [39], that could be responsible for the presence of JH. Another limitation is the small number of patients assessed with a low rate of female gender.

## 6. Conclusions

The results of our study suggest that JH could be considered as a clinical characteristic of ASD patients. Therefore, JH needs to be assessed in all ASD children in order to schedule a better rehabilitation program and to allow for the possibility of applying the research in practice.

Further studies with a higher number of children would shed light on the link between connective tissue, JH, and non-syndromic ASD. Furthermore, future quantitative MRI studies should focus on identifying possible morphological brain markers to clarify the neural networks sustaining the pathophysiology of JH and autism.

## Figures and Tables

**Table 1:** Mean ± SD score of the age, total intelligence quotient (IQ), and ADOS II score.

	AGE	Total IQ	Total ADOS II Score	AS Score	CRR Score	Total Severe Score
**Total sample (N = 67)**	6.00 ± 3.87	73.88 ± 18.15	16.12 ± 5,81	11.76 ± 4.77	4.22 ± 1.94	6.33 + 2.31
**Male (N = 62)**	5.90 ± 3.8	7.65 ± 18.65	17.06 ± 5.79	12.82 ± 4.69	4.21 ± 1.90	6.69 + 2.23
**Female (N = 5)**	7.24 ± 4.75	65.40 ± 7.83	15.2 ± 6.69	10.40 ± 6.02	4.80 ± 2.59	5.60 ± 3.36
**Range** **2–4 years (N = 30)**	2.41 ± 0.59	53.00 ± 14.65	19.13 ± 4.21	14.17 ± 3.73	4.83 ± 1.68	6.60 + 1.94
**Range ≥ 5 years (N = 37)**	8.32 ± 3.86	77.03 ± 19.89	14.30 ± 6.76	10.75 ± 4.95	3.55 ± 2.42	6.42 + 2.50

**Table 2:** Mean± SD of Beighton score in non-syndromic ASD children.

	Total Laxity Score	Right Thumb	Left Thumb	Right Fifth Finger	Left Fifth Finger	Right Elbow	Left Elbow	Right Knee	Left Knee	Right or Left Ankle/Touch Floor
**Total sample** **(N = 67)**	5.04 ± 2.76	0.93 ± 2.36	0.63 ± 0.71	0.81 ± 1.03	0.70 ± 0.46	0.79 ± 0.90	0.81 ± 1.23	0.34 ± 0.64	0.34 ± 0.48	0.61 ± 0.80
Male * **(N = 62)**	5.13 ± 2.79	0.97 ± 2.44	0.65 ± 0.73	0.82 ± 1.06	0.71 ± 0.46	0.81 ± 0.92	0.81 ± 1.28	0.35 ± 0.66	0.35 ± 0.48	0.65 ± 0.81
Female * **(N = 5)**	4.00 ± 2.35	0.4 ± 0.55	0.4 ± 0.55	0.66 ± 0.55	0.66 ± 0.55	0.66 ± 0.55	0.8 ± 0.45	0.2 ± 0.45	0.2 ± 0.45	0.2 ± 0.45
**Range** 2–4 years * (N = 30)	8.00 ± 3.00	0.93 ± 1.78	0.73 ± 0.78	0.90 ± 0.6	0.77 ± 0.43	0.77 ± 0.77	0.63 ± 0.49	0.27 ± 0.45	0.33 ± 0.48	0.87 ± 0.94
Range ≥ 5 years * (N = 37)	4.70 ± 2.54	0.92 ± 0.76	0.54 ± 0.65	0.73 ± 1.02	0.65 ± 0.48	0.81 ± 1.00	0.95 ± 1.60	0.41 ± 0.76	0.35 ± 0.48	0.41 ± 0.60

* No significant difference according to the age of assessment and gender (*p* > 0.05).

**Table 3:** Mean ± SD of Beighton score in control group children.

	Total Laxity Score	Right Thumb	Left Thumb	Right Fifth Finger	Left Fifth Finger	Right Elbow	Left Elbow	Right Knee	Left Knee	Right or Left Ankle/Touch Floor
Total sample * **(N = 67)**	0.97 ± 1.22	0.12 ± 0.33	0.15 ± 0.36	0.07 ± 0.25	0.07 ± 0.25	0.11 ± 0.32	0.15 ± 0.36	0.12 ± 0.33	0.1 ± 0.30	0.34 ± 0.52
**Male ** **(N = 62)**	0.97 ± 1.25	0.15 ± 0.36	0.08 ± 0.27	0.08 ± 0.27	0.12 ± 0.33	0.15 ± 0.36	0.14 ± 1.35	0.11 ± 0.31	0.11 ± 0.31	0.02 ± 0.12
**Female ** **(N = 5)**	1 ± 1	0.2 ± 0.45	0	0	0	0.20 ± 0.45	0	0	0	0.2 ± 0.45
Range * **2–4 years (N = 30)**	0.91 ± 1.09	0.25 ± 0.44	0.31 ± 0.47	0.11 ± 0.32	0.14 ± 0.35	0	0.02 ± 1.16	0	0	0.02 ± 0.94
Range ≥ 5 years * (N = 37)	1.02 ± 1.34	0	0	0.02 ± 0.16	0	0.22 ± 0.41	0.28 ± 0.45	0.25 ± 0.43	0.2 ± 0.4	0.02 ± 0.16

* Significant difference between ASD group and control group according to the age of assessment (*p* < 0.05).

## Data Availability

Data is contained within the article.
